# The association between body mass index and brain morphology in children: a population-based study

**DOI:** 10.1007/s00429-020-02209-0

**Published:** 2021-01-23

**Authors:** Cathelijne Steegers, Elisabet Blok, Sander Lamballais, Vincent Jaddoe, Fabio Bernardoni, Meike Vernooij, Jan van der Ende, Manon Hillegers, Nadia Micali, Stefan Ehrlich, Pauline Jansen, Gwen Dieleman, Tonya White

**Affiliations:** 1 Department of Child and Adolescent Psychiatry/Psychology, Erasmus Medical Center-Sophia Children’s Hospital, Rotterdam, The Netherlands; 2 The Generation R Study Group, Erasmus University Medical Center, Rotterdam, The Netherlands; 3 Department of Epidemiology, Erasmus MC, Rotterdam, The Netherlands; 4 Department of Clinical Genetics, Erasmus MC, Rotterdam, The Netherlands; 5 Department of Pediatrics, Erasmus University Medical Center, Rotterdam, The Netherlands; 6 Division of Psychological and Social Medicine and Developmental Neuroscience, Faculty of Medicine, Technische Universität Dresden, Dresden, Germany; 7 Department of Radiology and Nuclear Medicine, Erasmus MC, Rotterdam, The Netherlands; 8 Translational Developmental Neuroscience Section, Eating Disorder Research and Treatment Center, Department of Child and Adolescent Psychiatry, Faculty of Medicine, Technische Universität Dresden, Dresden, Germany; 9 Department of Psychology, Education, and Child Studies, Erasmus University Rotterdam, Rotterdam, The Netherlands; 10 Department of Psychiatry, Faculty of Medicine, University of Geneva, Geneva, Switzerland; 11 Division of Child and Adolescent Psychiatry, Department of Child and Adolescent Health, Geneva University Hospital, Geneva, Switzerland; 12 Great Ormond Street Institute of Child Health, University College London, London, UK

**Keywords:** BMI, Gyrification, Cortical folding, Cortical thickness, Brain development

## Abstract

Brain morphology is altered in both anorexia nervosa and obesity. However, it is yet unclear if the relationship between Body Mass Index-Standard Deviation Score (BMI-SDS) and brain morphology exists across the BMI-SDS spectrum, or is present only in the extremes. The study involved 3160 9-to-11 year-old children (50.3% female) who participate in Generation R, a population-based study. Structural MRI scans were obtained from all children and FreeSurfer was used to quantify both global and surface-based measures of gyrification and cortical thickness. Body length and weight were measured to calculate BMI. Dutch growth curves were used to calculate BMI-SDS. BMI-SDS was analyzed continuously and in two categories (median split). The relationship between BMI-SDS (range − 3.82 to 3.31) and gyrification showed an inverted-U shape curve in children with both lower and higher BMI-SDS values having lower gyrification in widespread areas of the brain. BMI-SDS had a positive linear association with cortical thickness in multiple brain regions. This study provides evidence for an association between BMI-SDS and brain morphology in a large sample of children from the general population and suggests that a normal BMI during childhood is important for brain development. Future studies could determine whether lifestyle modifications optimize BMI-SDS result in return to more typical patterns of brain morphology.

**Supplementary Information:**

The online version contains supplementary material available at 10.1007/s00429-020-02209-0.

## Introduction

The saying ‘you are what you eat’ also applies to our brains. Indeed, the brain is a unique organ, utilizing around 20% of the body’s energy (Herculano-Houzel 2011). During childhood development, the metabolism of the brain is over twice as high as in adulthood (Chugani 1998). Over the last decennia changes in eating behavior, with people choosing more unhealthy and higher caloric products, has resulted in a concomitant increase in body mass index (BMI) (Nagel et al. 2009; WHO 2017). This has led to questions regarding the impact this can have on the developing brain. Eating behavior has been shown to impact psychosocial and physical health (Grieken et al. 2013) and may influence brain morphology, but less is known about alterations in brain morphology associated with differences in BMI (Gustafson et al. 2004; Raji et al. 2010; Gregory et al. 2016; White et al. 2002; Dekkers et al. 2019), which is influenced by caloric intake.

Various studies have explored the relationship between BMI and global brain metrics, including cortical thickness (CT) and gyrification, the latter being a measure of the degree of cortical folding (Zilles et al. 1988; Armstrong et al. 1995). Gyrification and CT are both positively related to grey matter volume, which is involved in a broad range of brain functions and is associated with higher order cognitive functions (Kanai and Rees 2011). The ontogeny of CT and gyrification separate during prenatal life (White et al. 2010). The origins of the cortical surface of the brain begin to take form beginning at approximately six weeks of gestational age, which is associated with asymmetric division of neuronal precursor cells along the ventricular zone. The cell division results in one neuronal precursor cell and one neuronal cell, the latter which migrates in an inside out pattern to form the cortical layer. This process ends at approximately 24 weeks and all the cells have migrated to form the six-layered cortical surface. At 24 weeks the morphology of the brain is lissencephalic, having a smooth surface. The formation of the fissures and folds that are so characteristic of the human brain are initiated at the end of the second trimester, with the most rapid changes in gyrification taking place during the third trimester (White et al. 2010). While prenatal life shows the most robust development, both CT and gyrification undergo considerable changes throughout life. Interestingly, gyrification has much lower heritability rates in twin studies (White et al. 2002), suggesting that this measure is much more modifiable by environmental factors. Indeed, girls with acute anorexia nervosa (AN) have pronounced differences in gyrification, which resolves following restoration of weight to within a healthy range (Bernardoni et al. 2018). Thus, differences in CT may reflect a greater contribution of genetic factors contributing to the relationship between CT and BMI, whereas gyrification may be more driven by environmental factors, and thus more modifiable.

Studies in both children and adults with AN have shown reduced gyrification compared to patients with recovered AN and healthy controls (Bernardoni et al. 2018; Miles et al. 2018). Little is known about the association between a high BMI and gyrification, although one adult study found no relationship (Medic et al. 2016). Studies of the relationship between BMI and CT have primarily focused on adults (Medic et al. 2016; Bar et al. 2015) or the elderly (Gustafson et al. 2004; Raji et al. 2010; Buchman et al. 2005). In patients with AN, a reduced CT has been observed that remains even after controlling for BMI, suggesting a relationship between AN and CT over and above BMI. In studies including participants within normal BMI ranges and participants with obesity, a negative association between BMI and CT has been observed (Gustafson et al. 2004; Raji et al. 2010). Finally one study of healthy adults did not find an association between BMI and CT (Medic et al. 2016).

Surprisingly, to the best of our knowledge, no studies to date have assessed the relation between global brain metrics and BMI across the full BMI spectrum in children. Childhood is an important period for brain development, as events which take place during childhood can long-lasting effects on the brain (Ars et al. 2019). Generally, factors that influence brain development during prenatal and early life would be expected to have more global effects on the developing brain (Ars et al. 2019) as multiple processes are occurring in concert. However, since individual brain regions show temporal differences in peak maturation (Lenroot and Giedd 2006), global effects could become ‘unmasked’ at different times, dependent on regional differences in brain maturation. Thus, it is prudent for studies to focus not only on global associations, but also on specific regions that might either be more involved or may be unmasked at different stages of development.

Given the importance of nutrition on human development in general, and specifically for the brain, it was our goal to study the relationship between BMI and global brain morphology within a large population-based cohort of school-aged children. Based on the fact that for gyrification a positive association with BMI was observed in patients with AN and no associations were observed within the high BMI spectrum, we hypothesized a positive relationship with low BMI, which would then reach a plateau with increasing BMI. Based on earlier findings on the relationship between CT and BMI, we hypothesized an inverted-U shape relationship.

## Methods

### Participants

**Fig. 1:**
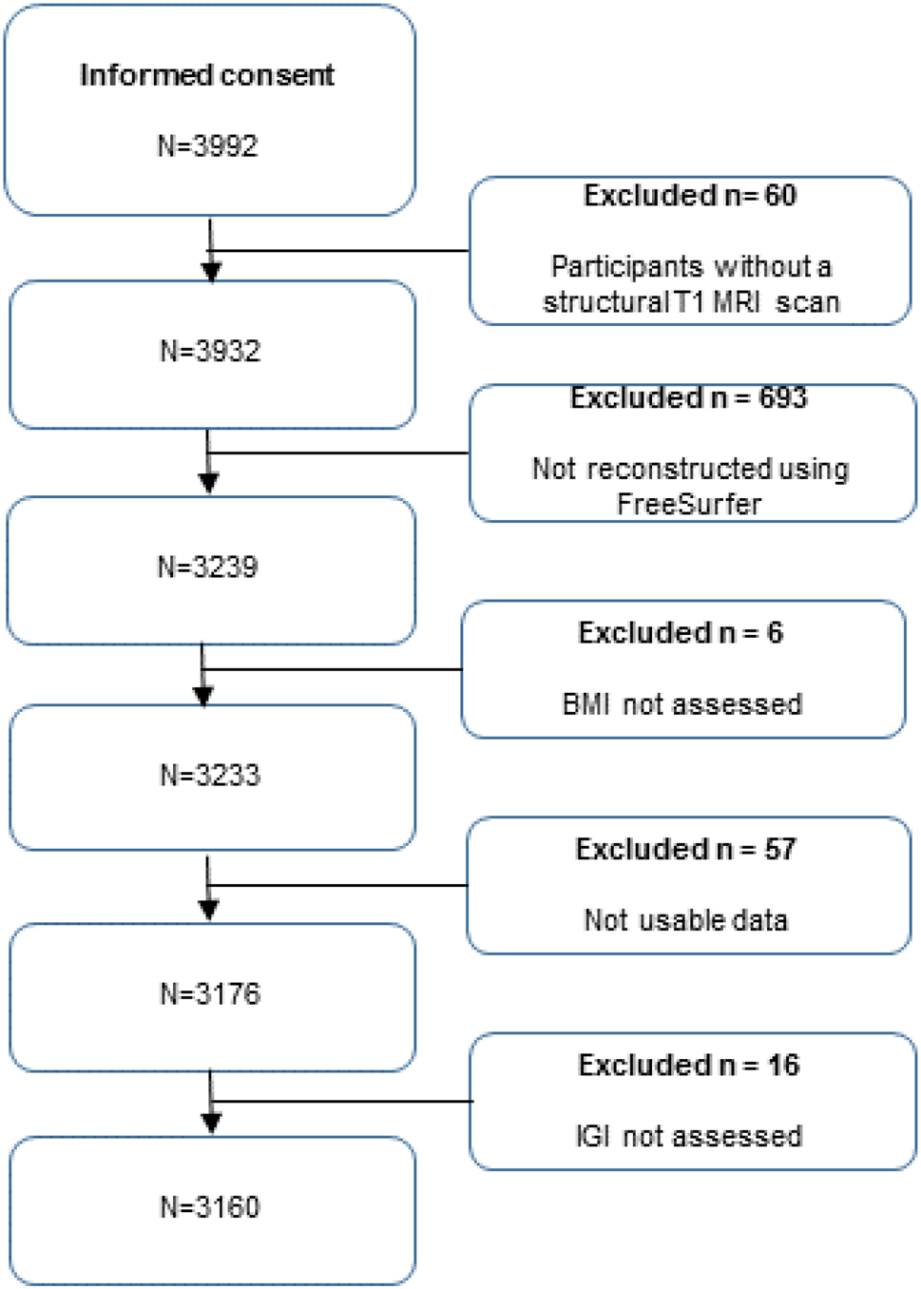
Flowchart in-/exclusion participants

This study was embedded within the Generation R Study, which is a prospective birth cohort in Rotterdam, the Netherlands (Jaddoe et al. 2012). The inclusion criteria for the initial recruitment included being pregnant, living within specific zip codes of Rotterdam, and having a planned delivery date between April 2002 until January 2006. After birth, the children have been followed up in multiple assessment waves. As part of the cohort's MRI study, 4087 children were scanned between March 2013 and November 2015 (White et al. 2018), processed structural T_1_-weighted images were obtained for 3932 of those. Children were excluded if BMI was not assessed (*n* = 6), if they had dental braces (*n* = 27), if incidental findings were found in the brain that significantly altered brain morphology (*n* = 16), if images failed reconstructions or had insufficient quality (*n* = 707), or if the gyrification index could not be calculated (*n* = 16). The final sample consisted of 3160 participants (50.3% female) (Fig. 1) who were 9-to-11 years of age (mean age at MRI = 10.1, SD = 0.6). Demographic information of the participants is provided in Table 1. The study was approved by the Medical Ethical Committee of the Erasmus Medical Centre in Rotterdam. Written informed consent was obtained from the legal representatives on behalf of the children.

**Table 1:** Baseline characteristics

	*n*	Demographic information
Age at BMI measurement	3160	9.80 (0.34)
Age at MRI measurement	3160	10.13 (0.59)
BMI-SDS (range − 3.82 to 3.31)	3160	0.26 (1.02)
BMI ≤ -1.3 SDS	168	5.3%
BMI ≥ 1.3 SDS	528	16.7%
IQ (M, SD)	2738	102.9 (14.83)
Sex		
Girl	1590	50.3%
Boy	1570	49.7%
Handedness		
Right	2751	92.3%
Left	315	10.5%
Missing	95	3.2%
Ethnicity of the mother		
Dutch	1796	56.8%
Other Western	378	12.0%
Non-Western	919	29.1%
Missing	67	2.1%
Education Level of the mother		
High	1541	48.9%
Middle	1174	37.2%
Low	187	5.9%
Missing	258	8.2%
Alcohol use of the mother		
Never drank in pregnancy	1045	33.0%
Drank until pregnancy was known	367	11.6%
Continued to drink in pregnancy occasionally	969	30.7%
Continued to drink in pregnancy frequently^a^	246	7.8%
Missing	533	16.9%
Smoking of the mother		
Never smoked during pregnancy	2138	67.7%
Smoked until pregnancy was known	246	7.8%
Continued smoking in pregnancy	370	11.7%
Missing	406	12.8%
CBCL mother report	2686	16.88 (14.83)

Values are frequencies for categorical measures, means and standard deviations for continuous measures

*MRI* magnetic resonance imaging, *BMI-SDS* Body Mass Index-Standard Deviation Score, *IQ* intelligence quotient, *CBCL* Child Behavior Check List

^a^Frequent continued alcohol use is defined as one or more glasses of alcohol per week in at least two trimesters

### Measures

#### Body Mass Index

Since the adult BMI categories do not correspond to children’s nutritional status at specific ages, we utilized an age and sex adjusted measure of BMI, which is commonly applied and termed the BMI-SDS (Zannolli and Morgese 1996). To adjust BMI for age, Dutch growth curves of the “Toegepast Natuurwetenschappelijk Onderzoek” (TNO) were used, which are based on WHO cut offs of − 1.0, − 2.0 and − 3.0 SD’s for malnutrition, corresponding roughly to 90%, 80% and 70% of expected weight for height (Cole et al. 2007). We used ≤ − 1.3 BMI-SDS as a cut-off to define children who were underweight and ≥ 1.3 as the cut-off to define children who were overweight. These cut offs correspond roughly to the upper and lower 10th percentile per age, and these cut-offs have been used in other studies evaluating AN (e.g. Seidel et al. 2017). BMI measurement was taken at a mean age of 9.8 years (SD = 0.34). Child body height and weight were measured in a dedicated research center by trained staff (Jansen et al. 2012). Height in standing position was measured with a stadiometer (Holtain Limited). Weight was measured using an electronic scale (SECA) without heavy clothing and shoes. BMI-SDS was calculated based on height and weight in kilograms/meter^2^ and adjusted for sex and age according to Dutch growth curves (Fredriks et al. 2000).

#### MRI data acquisition

The mean age on which MRI measurement took place was 10.13 years (SD = 0.59 years). All MRI images were acquired on a single 3.0 T GE 750w MR system (General Electric Healthcare, Milwaukee, WI, USA) utilizing an eight-channel head coil. T_1_-weighted structural images were obtained with an inversion recovery-prepared fast spoiled gradient recalled sequence in a 3D-acquisition [TR = 8.77 ms, TE = 3.4 ms, TI = 600 ms, NEX = 1, flip angle = 10°, field of view (FOV) = 220 × 220 mm, number of slices = 230, resolution = 1.0 mm^3^] (White et al. 2013).

#### Image processing and quality assessment

T_1_-weighted images were processed using FreeSurfer Software, version 6.0 (http://surfer.nmr.mgh.harvard.edu). The technical details of these procedures are described elsewhere (Muetzel et al. 2019). In brief, this included removal of the non-brain tissue, Talairach transformation, segmentation of white and grey matter structures, tessellation of the grey-white matter boundary, topology correction and surface deformation to identify the cortical grey-white matter boundary and the grey-cerebrospinal fluid boundary. Individual brain voxels were labeled as white matter, grey matter or cerebrospinal fluid (CSF). The FSaverage brain surface template was used to reconstruct and analyze surface-based cortical morphometry. For global analyses, we used FreeSurfer metrics of mean CT and the mean local Gyrification Index (mean lGI) over the entire brain (mean value of all cortical). Since the left and right hemisphere were highly correlated (GI: *r* = 0.92, *p* < 2 × 10^16^; CT: *r* = 0.77, *p* < 2 × 10^16^) and since we did not hypothesize lateralized effects of the global measures, we created global measures for lGI and CT by weighting the means of both hemispheres.

For surface-based analyses, CT was calculated as the closest distance from the grey-white matter boundary to the grey-cerebrospinal fluid (CSF) boundary at each vertex on the tessellated surface (Fischl and Dale 2000). The labeling of clusters identified in the brain was performed using the technique of Klein and Tourville (2012). The lGI was calculated, using the method of Schaer et al. (2008) based on a 3D spherical extension of the gyrification index defined by Zilles et al. (1988). Prior to the surface-based analyses images were smoothed using a 10 mm full-width-at-half-maximum Gaussian kernel for CT analyses and 5 mm for analyses involving the lGI. Reconstructed images were visually inspected and rated using a three-point Likert scale including unusable to poor, fair to good, and very good to excellent. Images that were rated as poor were excluded (White et al. 2013).

### Covariates

Multiple parental and child characteristics were considered as covariates. Maternal ethnicity was based on the country of birth from the parents and was subdivided in Dutch, Other Western (American Western, Asian Western, European, Oceania & Indonesian) and Non-Western (African, American Non-Western, Asian Non-Western, Cape Verdean, Dutch Antilles, Moroccan, Surinamese & Turkish). Maternal education was included as a proxy for SES. The level of education of the mother was divided into: Low (no education finished and primary education finished), Middle (secondary school or lower vocational training) and High (Higher vocational training or University degree). Information on maternal smoking and drinking during pregnancy was collected prenatally. Handedness was measured using the Edinburgh Handedness Inventory (EHI) (Oldfield 1971).

Non-verbal IQ of the child was assessed when the children were 5–8 years of age using the abbreviated version of non-verbal intelligence test (Snijders-Oomen Niet-verbale intelligentie test-Revisie, SON-R 2.5–7) (Tellegen et al. 2005). We used two subsets: Mosaics, which assesses spatial visualization abilities, and Categories, which assesses abstract reasoning abilities. After the raw scores were standardized, they were converted into the SON-R IQ score using age-specific reference scores. Finally, the total score of the Child Behavior Checklist (CBCL), a reliable and valid questionnaire to assess behavioral problems (Achenbach and Rescorla 2003), was included as a covariate. The CBCL consists of 113 questions using a three-point Likert scale (0 = not true, 1 = somewhat true, 2 = very true). In a sensitivity analysis we tested whether the amount of physical activity affects the association between BMI-SDS and mean lGI. Physical activity was assessed by asking parents in a questionnaire how much time their children play outside per week.

### Statistical analyses

Statistical analyses were performed using R version 3.6.3 (Team RC 2015). The association between BMI-SDS and the three measures of brain morphology were examined with linear regression analyses. We examined whether a linear term best explained the relationship between BMI and brain morphology or whether the addition of squared or cubic polynomials would improve the fit. Model fit was compared using an ANOVA. The quadratic model provided the best fit for mean lGI and the linear model for global CT (see supplementary Table 1 for relationships between the different models).

We tested four models with increasing numbers of covariates. In our first model we adjusted for sex, age and handedness, the second model we added maternal education, ethnicity, smoking and drinking during pregnancy and child IQ as covariates, the third model was additionally adjusted for child behavior. To assess whether lGI and CT are associated with BMI-SDS in specific brain regions, we entered mean lGI and global CT in a fourth model. Correction for multiple testing was performed using the Benjamini–Hochberg approach (Benjamini and Hochberg 1995) for the two global variables (mean lGI, and mean CT).

We observed a quadratic relationship between BMI-SDS and mean lGI with a peak around the median BMI-SDS. As we did not want to assume that both high and low BMI involved the same brain regions, we performed a median split to assess local gyrification in groups with low and high BMI-SDS. We analyzed the association between BMI-SDS and local gyrification in both groups using linear regression models with local gyrification as the dependent and BMI-SDS as the independent variable. For CT we did not use this split, since global CT showed a linear relationship with BMI-SDS.

Vertex-wise analyses were performed in R using the QDECR package (https://github.com/slamballais/QDECR). Surface-based analyses were corrected for multiple testing using Gaussian Monte Carlo Simulations with a cluster-wise correction. The cluster-forming threshold was set to *p* = 0.001, as it has shown to correspond closely to a false-positive rate of 0.05 (Greve and Fischl 2018). The tests were additionally Bonferroni corrected to account for both hemispheres (i.e. *p* < 0.025 cluster-wise).

## Results

### Demographics

Demographic characteristics and measures of brain morphology are shown in Table 1. A distribution of our BMI-SDS variable is shown in supplementary Fig. 1. Boys were slightly older at the time of the MRI (mean difference (MD) = 0.05 years, *t* = 2.85, *df* = 3907.5.8, *p* = 0.0045) and at the time of the BMI-SDS measurement (mean difference = 0.02 years, *t* = 2.098, *df* = 3865.6, *p* = 0.036). The CBCL sum score was higher in boys than in girls (MD = 2.24, *t* = 3.92, *df* = 2669.3, *p* = 8 × 10^–5^). The mean lGI (MD = 0.07, *t* = 22.049, *df* = 3154.2, *p* < 2.2 × 10^–16^) was higher in male participants than female participants. A negative correlation between mean lGI and age was found [*r*(3158) = − 0.08, *p* < 5.24 × 10^–6^].

Supplementary Table 2 shows the demographic characteristics of the participants in the low and high BMI-SDS subgroups. Mothers of participants with a lower BMI-SDS were more often of Dutch nationality (*χ*^2^ = 84.57, *df* = 2, *p* ≤ 2.2 × 10^–16^), had higher education (*χ*^2^ = 98.578, *df* = 2, *p* ≤ 2.2 × 10^–16^), and smoked (*χ*^2^ = 7.93, *df* = 2, *p* = 0.02) and used more alcohol (*χ*^2^ = 50.61, *df* = 3, *p* = 5.94 × 10^−11^) during pregnancy. In addition, participants in the low BMI-SDS group were slightly younger (MD = 0.02, *t* = − 2.68, *df* = 3151, *p* = 0.007) and had a higher non-verbal IQ (MD = 3.05, *t* = 5.42, *df* = 2736, *p* = 6.54 × 10^–8^) compared to the participants in the high BMI-SDS group.

A non-response analysis evaluating the differences between children included and excluded showed that the non-responders more often came from families with a middle education level (*χ*^2^ = 16.327, *df* = 2, *p* = 0.0003), had a lower non-verbal IQ (MD = 1.98, *t* = − 2.96, *df* = 878.85, *p* = 0.003), had a lower GI (MD = 0.058, *t* = − 9.91, *df* = 842.39, *p* ≤ 2.2 × 10^–16^) and came from mothers who drank less during pregnancy (*χ*^2^ = 8.6931, *df* = 3, *p* = 0.03), than those who were included in the study.

### Continuous Measures of BMI-SDS with global (mean lGI) and local Gyrification (lGI)

**Fig. 2:**
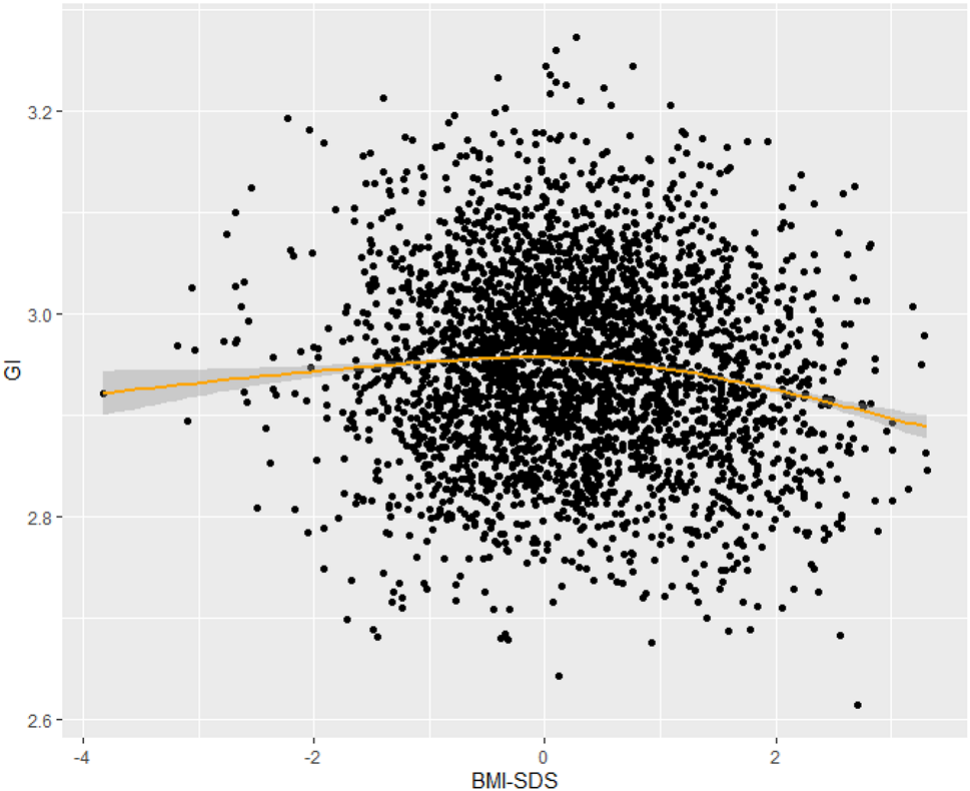
Polynomial regression of BMI-SDS and global gyrification

Results for the associations of BMI-SDS with the measure of global gyrification (mean lGI), are displayed in Table 2. When fitting the mean lGI data to a quadratic polynomial in BMI-SDS (Fig. 2) we found that the association was significant (*B* = − 0.003, *p* = 0.0042) in model 3. After removing participants with very low and very high BMI-SDS (BMI-SDS ≤ 1.3 and BMI-SDS ≥ 1.3), both the associations between BMI-SDS and mean lGI were no longer significant, suggesting that it was the ends of the spectrum that were driving the differences. In a sensitivity analyses we investigated whether the average amount of outdoor play time per week affects the association between mean lGI and BMI-SDS, by adding this variable to our second model. We found that outdoor play time was not significantly associated with BMI-SDS and that the association between mean lGI and BMI-SDS remained essentially the same (*B* = − 0.003, *p* = 0.003). In a second sensitivity analyses we stratified our analyses of BMI-SDS and mean lGI by sex in our second model. While the relationship between BMI-SDS and mean lGI was significant in boys (*B* = − 0.003, *p* = 0.02), but not in girls (*B* = − 0.003, *p* = 0.06), the effect estimates were similar between boys and girls and thus power may have played a role.

**Table 2:** The association of global Gyrification Index (mean lGI) and body mass index-standard deviation score (BMI-SDS)

Measurement	Model	Factor	B for BMI-SDS	SE	*t* value	*p*
Mean local Gyrification Index	Model 1					
		Intercept	3.23	0.028	116.17	< 2 × 10^16^**
		BMI-SDS^2^	− 0.006	0.001	− 5.32	0.0000001**
	Model 2					
		Intercept	3.13	0.031	102.44	< 2 × 10^16^**
		BMI-SDS^2^	− 0.003	0.001	− 2.98	0.0029*
	Model 3					
		Intercept	3.14	0.031	102.40	< 2 × 10^16^**
		BMI-SDS^2^	− 0.003	0.001	− 2.86	0. 0042*

Mean local Gyrification Index and quadratic models for BMI-SDS. B's are averaged from 100 imputed datasets. Model 1 is adjusted for sex, age and handedness. Model 2 is additionally adjusted for education and ethnicity of the mother, maternal smoking and drinking during the pregnancy and the IQ of the child. Model 3 is additionally adjusted for behavior of the child. All tests survived correction using the Benjamini Hochberg correction for multiple testing with an alpha = 0.05

*Significant at *p* < 0.05

**Significant at *p* < 0.001

To further study the association between BMI-SDS and lGI, we applied a median split to the data using a split value of 0.21 BMI-SDS. Table 3 shows the relationships between BMI-SDS < 0.21 (low BMI subgroup) and the lGI and the relationship between BMI-SDS ≥ 0.21 (high BMI subgroup) and the lGI. As expected, in the low BMI subgroup we observed that the lGI increases with higher BMI-SDS, whereas in the high BMI-SDS subgroup we observe a decrease in lGI with an increase in BMI-SDS. Clusters that remained significant after multiple testing correction were annotated to the brain region in which the largest percentage of the cluster was located (for a more detailed description of cluster annotation, see Supplemental Table 4 for lGI and Table 5 for CT). For the low BMI-SDS group, we observed a statistically significant increase in lGI in BMI-SDS and a decrease in the lGI in the right paracentral gyrus (*p* = 0.0001) and the left posterior cingulate gyrus (*p* = 0.00024) in model 1. These clusters did not remain significant after additional adjustment in model 2, 3 and 4.

**Table 3:** The association of the body mass index (BMI-SDS) and local Gyrification Index (lGI)

Weight status	Model	Hemisphere	Anatomical region	Area size (mm^2^)	MNI			Cluster-wise unstandarized Beta coefficient
					*x*	*y*	*z*	
Low	Model 1							
		RH	Paracentral	711.51	10.5	− 8.5	41.2	0.023
		LH	Posterior cingulate	761.95	− 18.3	− 31.6	39.7	0.027
High	Model 1							
		RH	Rostral middle frontal	32,427.06	23.0	59.9	5.6	− 0.043
			Precuneus	3026.13	25.7	− 60.7	7.5	− 0.037
			Paracentral	1092.04	7.8	− 29.2	50.5	− 0.023
		LH	Precentral	30,457.85	− 60.1	− 9.2	15.3	− 0.053
			Rostral middle frontal	7049.75	− 21.3	55.3	5.9	− 0.023
	Model 2	RH	Rostral middle frontal	1605.64	36.8	49.0	− 28.7	− 0.024
			Middle temporal	1249.96	65.2	− 28.6	− 13.9	− 0.045
			Temporal pole	380.94	33.6	3.0	− 9.3	− 0.021
		LH	Precentral	4248.58	− 59.6	− 10.0	32.6	− 0.045
			Superior temporal	797.47	− 60.0	− 8.0	− 1.5	− 0.053
			Superior temporal	350.82	− 54.5	− 30.7	− 0.9	− 0.053
	Model 3	RH	Rostral middle frontal	1581.90	36.8	49.0	− 9.3	− 0.024
			Middle temporal	1175.52	65.2	− 28.6	− 13.9	− 0.045
			Temporal pole	377.33	33.9	2.8	− 28.3	− 0.021
		LH	Postcentral	4018.12	− 59.6	− 10.0	32.6	− 0.045
			Superior temporal	692.87	− 60.0	− 8.0	− 1.5	− 0.053
	Model 4	LH	Postcentral	362.73	− 59.6	− 10.0	32.6	− 0.035

Model 1 is adjusted for gender, age and handedness. Model 2 is additionally adjusted for education and ethnicity of the mother, maternal smoking and drinking during the pregnancy and the IQ of the child. Model 3 is additionally adjusted for behavior of the child. Model 4 is further adjusted for global gyrification

Correction for multiple testing was performed using randomize

**Fig. 3:**
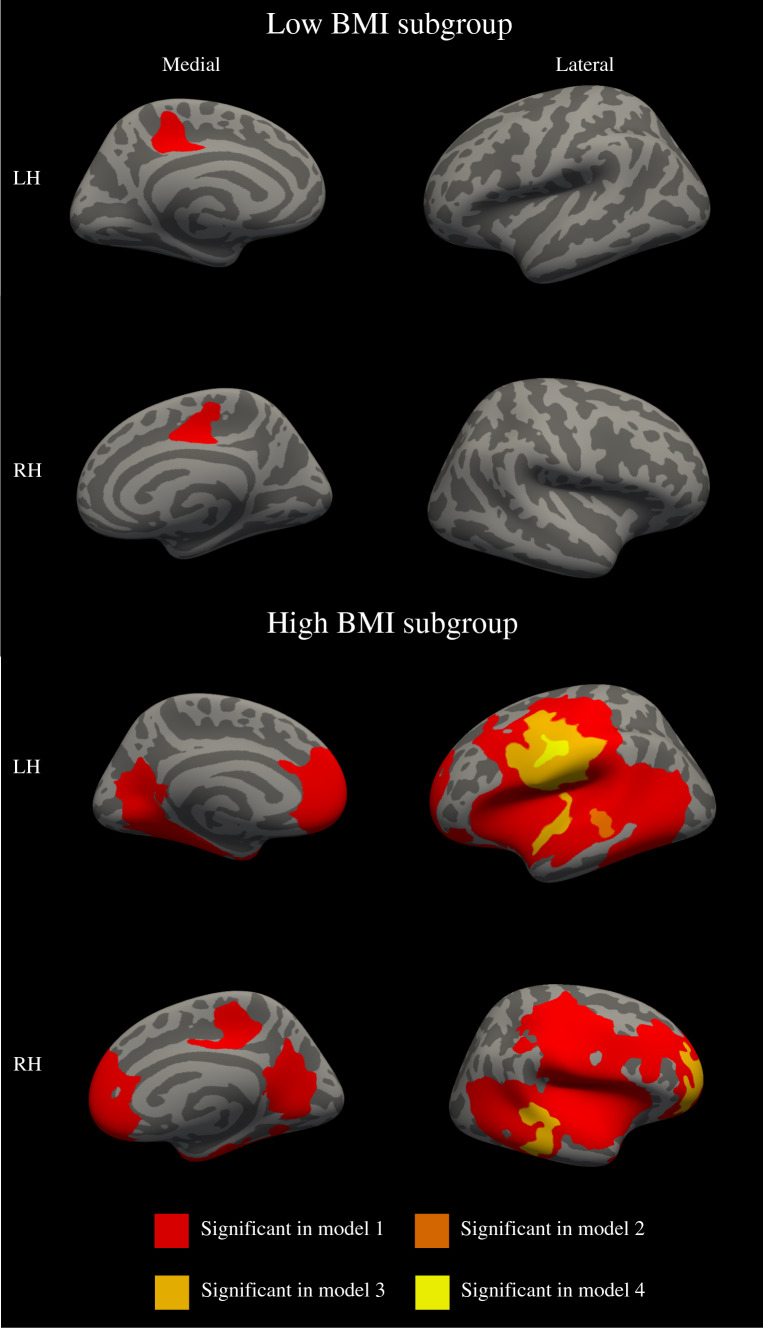
BMI-SDS and the local gyrification Index

In the high BMI-SDS subgroup we observed significant clusters in all models. A positive relationship between BMI-SDS and the lGI was observed in clusters in the right middle temporal sulcus (*p* = 0.0001), rostral middle frontal gyrus (*p* = 0.0001), and temporal pole (*p* = 0.016), and the left postcentral (*p* = 0.0001), and in the left superior temporal gyri (*p* = 0.001) (Fig. 3). After controlling for global gyrification (model 4) to test for specificity of the finding, only the left postcentral gyrus remained significant (*p* = 0.0098).

### Continuous measures of BMI-SDS with global and local cortical thickness

Assessing global cortical thickness, we found a significant linear association between BMI-SDS and global CT (Table 4) with a higher BMI-SDS associated with higher global CT. Consequently, in a sensitivity analysis, we stratified this analysis by sex. BMI-SDS was significantly associated with global CT in both boys (*B* = 0.007, *p* = 0.0002) and girls (*B* = 0.005, *p* = 0.006).

**Table 4:** The linear association of cortical thickness (CT) and body mass index (BMI-SDS)

Model	Factor	*B*	SE	*t* value	*p*
Model 1					
	Intercept	2.82	0.024	117.29	< 2 × 10^–16^**
	BMI-SDS	0.005	0.001	3.06	0.00008**
Model 2					
	Intercept	2.86	0.027	105.35	< 2 × 10^–16^**
	BMI-SDS	0.006	0.001	4.68	0.000003**
Model 3					
	Intercept	2.86	0.027	105.00	< 2 × 10^–16^**
	BMI-SDS	0.006	0.001	4.68	0.000003**

Linear regression analysis of BMI-SDS and CT. B's are averaged from 100 imputed datasets. BMI-SDS values are centered around the mean. Model 1 is adjusted for sex, age and handedness. Model 2 is additionally adjusted for education and ethnicity of the mother, maternal smoking and drinking during the pregnancy and the IQ of the child. Model 3 is additionally adjusted for behavior of the child. All measurements survived correction using the Benjamini Hochberg correction for multiple testing with the alpha = 0.05

*Significant at *p* < 0.05

**Significant at *p* < 0.001

**Fig. 4:**
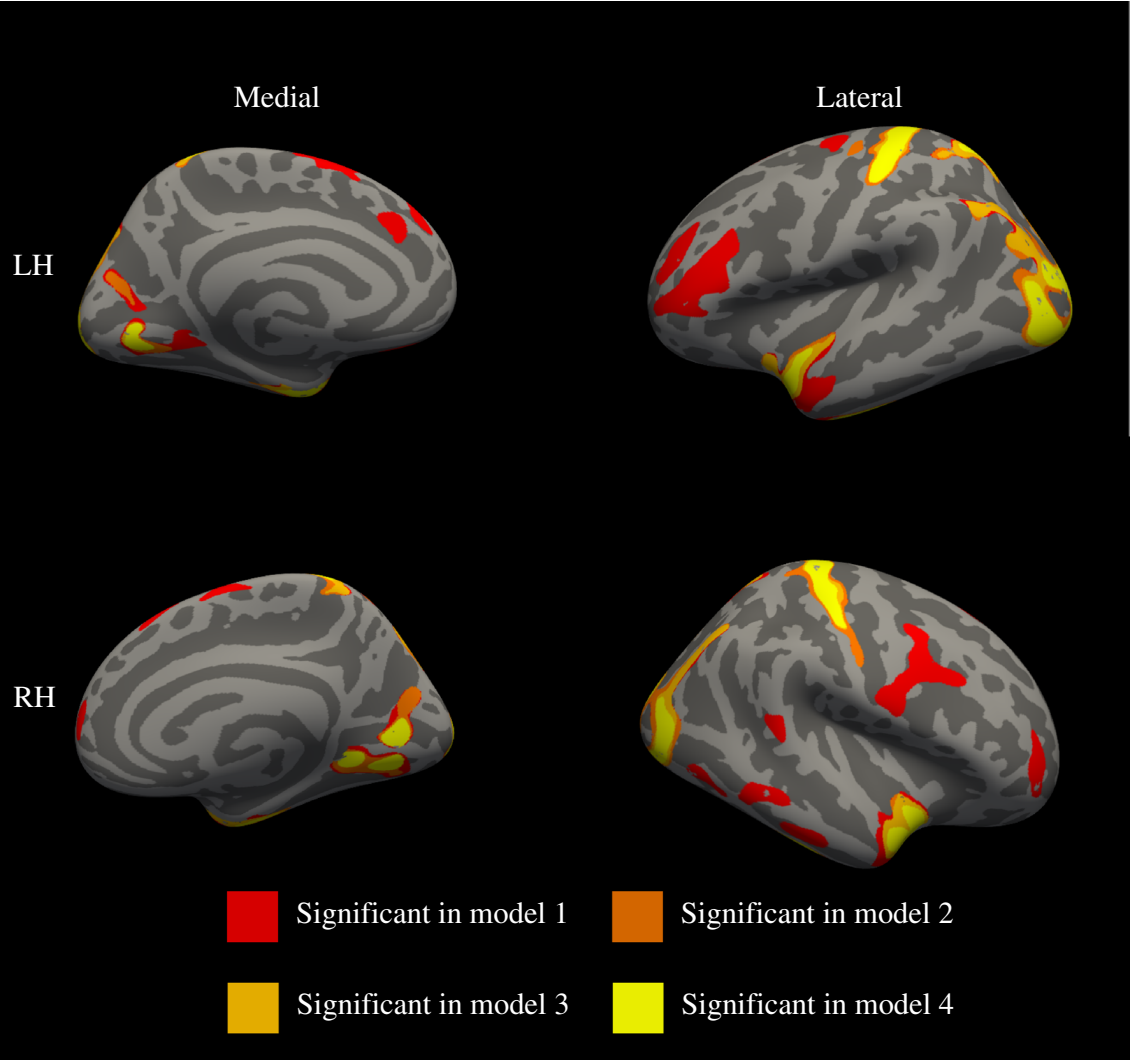
BMI-SDS and cortical thickness

Assessing the association between BMI-SDS and local CT, we found positive associations between BMI-SDS and local CT in all three models (Supplementary Table 3). Results from model 3 showed one cluster in the right superior parietal (*p* = 0.0001), one in the left and right (*p* = 0.0001) superior temporal, one in the right inferior temporal (*p* = 0.0001), one in the right pericalcarine (*p* = 0.0003), one in the left and one in the right (*p* = 0.0001) lateral occipital, one in the left and one in the right postcentral (*p* = 0.0001), one in the left (*p* = 0.0002) and two in the right lingual (*p* = 0.0001 and *p* = 0.004), and one in the left superior parietal gyri (*p* = 0.0001) (Fig. 4).

## Discussion

Examining the relationship between the continuum of BMI-SDS and gyrification, we found evidence for an inverted-U shaped curve, with reduced gyrification in both children with a low and high BMI-SDS. Moreover, we found a positive linear relationship between BMI-SDS and global CT. Vertex-wise analyses showed associations between BMI-SDS and both lGI and local CT in widespread areas of the brain. Our findings add important information to the current knowledge of the relationship between cortical morphology and BMI for at least two reasons. First, the observed relationship between the broad continuum of BMI-SDS and gyrification in a pediatric population-based study of school-aged children, suggesting an underlying neurobiological relationship. Second, BMI-SDS may be an important covariate to integrate into studies that assess the surface morphology of the brain. This is especially true in longitudinal measures of psychiatric disorders in which medications, such as psychotropic medication, can result in an increase in BMI (Upadhyay et al. 2019).

### The association between the BMI-SDS and global gyrification

We found an inverted-U relationship between BMI-SDS and global gyrification, with lower global gyrification in children with both lower and higher BMI-SDS. At the lower end of the spectrum, the relationship we observed is very similar to what has been found in patients with AN, namely lower gyrification with lower BMI. Research involving patients with AN focus on the relationship between abnormally low versus those with typical BMI. Interestingly, girls with severely low BMI have significant and widespread decreases in global gyrification which resolves after weight restoration (Bernardoni et al. 2018). However, we do not know whether the children with very low BMI in the general population will normalize following weight restoration.

We also observed a negative relationship between BMI and gyrification at the higher end of the BMI spectrum, which is in contrast to an adult sample that did not observe an association between BMI and gyrification (Medic et al. 2016). Since childhood is an important period for brain development (Lenroot and Giedd 2006) and particularly for gyrification and cortical complexity (Aleman-Gomez et al. 2013; White et al. 2010; Magnotta et al. 1999), one reason for the discrepant findings could be that the association between BMI and brain morphology is developmental, and thus studies during childhood do not parallel those in adults. Another explanation could be that genetic factors, not accounted for in this study, are responsible for the low gyrification in the high BMI group. However, one of the reasons that we selected gyrification is that it is less driven by genetic factors (Lohmann et al. 1999), as was shown in our earlier study of girls with AN (Bernardoni et al. 2018), and thus may be a better marker for environmental factors, such as diet.

We found evidence for a graded increase in global gyrification from low BMI-SDS to normal BMI-SDS children. Interestingly, our findings also suggest that the relationship flips, reflecting a negative relationship between normal and high BMI-SDS. Due to the cross-sectional design, we are unable to infer causality. Interestingly, the association between BMI-SDS and global gyrification was no longer significant after removing the participants with an extreme low and extreme high BMI-SDS from our dataset, suggesting that our findings are driven largely by the individuals with more extreme high and low BMI-SDS. To some extent this is reassuring, as it implies that most individuals within a wide normal range of BMI-SDS have no difference in gyrification attributable to BMI. What is unclear is whether those individuals in the extremes of the population have other factors that may be driving the gyrification differences. Future studies could determine whether lifestyle modifications for those with an extreme low (separate from those with AN) or high BMI will result in more typical patterns of brain morphology. Longitudinal and interventional studies of those with high BMI are needed to address the question about causality.

### The association between BMI-SDS and local gyrification

Studying regional differences in cortical surface morphology, we found associations between BMI-SDS and gyrification in specific brain regions. However, when also correcting for global gyrification, only the left cluster in the postcentral gyrus remained significant in the high BMI-SDS group. Thus, many of the regions we identified could be considered global differences, whereas the postcentral gyrus may be a localized region that has greater specificity for the effect of BMI-SDS on gyrification. Since controlling for outdoor play time, a measure indicative of physical activity, did not change the results, it is less likely that this finding is due to differences in physical activity between children. A higher left paracentral surface area in patients with normoglymenic obesity has been documented earlier by Bernardes and colleagues (Bernardes et al. 2018), and thus this brain area may show greater specificity in relation to BMI. Additional studies, preferably longitudinal or interventional studies are needed to confirm the specificity of this finding. In general, our findings support that the relationship between BMI-SDS and gyrification tend toward global differences.

### The association between BMI-SDS and CT

The positive relationships we observed between BMI-SDS and CT in both local and global analyses are consistent with findings observed by Lavagnino et al. (2016), who found a positive relationship between BMI and CT, although their results did not survive correction for multiple comparisons. Their sample size consisted of 21 patients with AN and 18 controls, so lack of power may be why their findings did not hold after correction for multiple comparison. Other studies, however, have shown a negative relationship between BMI-SDS and CT (Gustafson et al. 2004; Raji et al. 2010; Lavagnino et al. 2018). Medic et al. found no global relationship between BMI and CT in a sample of individuals with obesity; however, they did find thinner cortices in specific regions, including the lateral occipital cortex and the ventromedial prefrontal cortex. In line with these findings, Fuglset et al. found no global association between BMI and CT, but reduced CT bilaterally in the superior parietal gyrus and in the right inferior parietal and superior frontal gyri in their sample of patients with AN. An important note is that the majority of those patients were partially weight-restored. There are also studies that report no association between BMI and CT, but these studies evaluate relationship between visceral fat and CT (Kaur et al. 2015; Saute et al. 2018) and BMI and grey matter volume in adolescents and adults (Weise et al. 2019; Caunca et al. 2019).

Our study investigated the relationship between the ages of nine to eleven, thus prior to, or in early stages of puberty. A possible explanation that would be interesting to investigate is the role of leptin. Leptin is a pleitropic hormone that can cross the blood–brain barrier and act on many brain areas. This could lead to inhibition of cell death (Fujita et al. 2002), which in turn could lead to a decrease in cortical thinning. CT typically increases during childhood and peaks during late childhood and early adolescence (Shaw et al. 2008; Giedd et al. 2012; Nie et al. 2013), thus a possibility is that leptin results in a slower decrease in the developmental trajectory of CT.

### Linear and quadratic associations and brain morphometry

Interestingly, whereas we found a quadratic association between BMI-SDS and global gyrification, the association between BMI-SDS and global CT was linear. Earlier neuroimaging studies focused primarily on clinical populations, where only one tail of distribution is investigated. However, with a large population-based sample, we are able to test both linear and non-linear models. Evidence from previous research shows that the development of CT is highly driven by genetic factors. Gyrification, while also driven by genetic factors, also is much more influenced by environmental factors. For example, the study of Bernardoni and colleagues (Bernardoni et al. 2018) showed that gyrification in individuals with AN is decreased, but is restored and almost back to normal after weight restoration. This was not true for cortical thickness. Thus, differences in CT may be an endophenotype of BMI, defining more a trait characteristic of an individual. Alternatively, gyrification may be more related to a state characteristic, and thus modifiable environmental factors, such as diet. It is possible that a poorer nutritional status has a negative effect on gyrification, which then explains the inverted-U shape association.

### Clinical implications

Alterations in gyrification may have several clinical implications. Since gyrification has been shown to be modifiable by interventions in women with AN, it is possible that altering diet or increasing exercise could alter cortical morphology in a wider population. Gyrification of the fetal brain develops primarily during the third trimester of pregnancy (Armstrong et al. 1995) and has been shown to decrease during adolescence (Aleman-Gomez et al. 2013; White et al. 2010). Disruptions in the development of gyrification is thought to reflect differences in the underlying brain components (i.e., neurons, synapses), potentially reflecting less efficient neural processing (Essen 1997). Disruptions in gyrification are associated with multiple psychiatric disorders, including autism (Blanken et al. 2015), obsessive compulsive disorder (Rus et al. 2017; Fan et al. 2013; Venkatasubramanian et al. 2012), schizophrenia (White and Hilgetag 2011), depression (Schmitgen et al. 2019) and Prader-Willi syndrome (Lukoshe et al. 2014). In most cases, psychopathology results in global or regional decreases in gyrification, thus gyrification abnormalities may also reflect nutritional factors and not exclusively the underlying neuropsychiatric disorders (White and Gottesman 2012).

Longitudinal studies that span childhood to adulthood will be important to better understand the relationship between psychopathology and cortical morphology, however, our findings suggest that BMI-SDS should be used as a covariate in pediatric neuroimaging studies, especially involving psychopathology. This is especially true for disorders such as schizophrenia, where medication is associated with increased weight gain.

## Strengths and limitations

There are a number of strengths of our study. First, the study was performed in a large, population-based study of child development in which the participants were recruited during prenatal life or at birth, which reduces selection bias. As a result, the participants entered the study prior to developing any psychopathology, such as eating disorders, anxiety, or depression. Our sample size is large, which is necessary in a population-based study to have sufficient participants in the tails of the distribution. Our study also has several limitations. As a cross-sectional study, we can only speculate on potential causal relationships. We show the presence of a relationship between BMI-SDS and GI and CT, however, we are unable to state anything about the temporal trajectories of these relationships. However, the BMI-SDS collected at the 5–6-year-old wave of Generation R had a Pearson Correlation of 0.8, suggesting that BMI-SDS is quite stable during middle- to late-childhood. Second, BMI-SDS was not assessed at exactly the same time as the MRI session. Despite controlling for the age at the time of scanning, it could be that the BMI-SDS changed over time. However, the time difference between the BMI measurements and MRI were relatively close (median = 0.1 month, Q1 = 0.04, Q3 = 0.27 months). Third, our study was performed in the general population and not in a clinical population. While this is also a strength of the study, our findings may not translate to clinical conditions such as AN. More research combining both clinical and general populations using longitudinal designs should be performed to better assess the temporal relationship in clinical, sub-clinical, and population differences in under- and overweight children. Fourth, although the largest part of our sample is likely to be pre-pubescent, we acknowledge that pubertal timing can influence BMI. Future studies should focus on whether puberty effects the relationship between BMI and brain structure. Fifth, since the brain undergoes considerable development from infancy into adulthood, some studies have applied study specific templates to better account for the developmental changes (Janssen et al. 2009). Since we used the FSaverage FreeSurfer template, which is based on adults, some of the age-related variability may not be fully captured. While age-specific templates are well matched for the age and characteristics of the participants, there is also evidence that once children have exceeded six years of age the growth characteristics of the brain are such that adult templates can be applied (Vân Phan et al. 2018). Further, one of the other advantages of using a standard commonly used template, such as FreeSurfer’s FSaverage, when children are old enough, is that it offers a level of standardization between different studies. We have consistently used the FSaverage brain in the Generation R Study (White et al. 2018), including studies in our earlier neuroimaging wave, which recruited children between six-to-nine years of age (Blanken et al. 2015; Mous et al. 2014). In addition, the large ABCD Study in the US, which matches the age group in our sample, applied a standard template that was created using T_1_-weighted images from 500 adults (Hagler et al. 2019). Sixth, while our goal is to study BMI within the general pediatric population, including the extreme tails of underweight and overweight, these may not necessarily equate with clinical diagnosis of anorexia nervosa or obesity. The goal is to assess clinical diagnoses in the future, once the children reach late adolescents, however this data has yet to be collected. Finally, some demographic measures differed between the participants who were included and excluded, so there may be some limitations to the generalizability of our findings.

## Conclusion

In conclusion, our study provides evidence for an association between BMI and cortical morphology in a large sample of school-aged children drawn from the general population. The relationship between BMI and gyrification has an inverted-U shaped distribution and is driven primarily by global differences in gyrification. Cortical thickness is positively associated with BMI. This study provides evidence that a normal BMI during childhood is associated with more typical measures of brain surface morphology, which may equate with more optimal brain development. Future studies should longitudinally assess the interplay between high/low BMI and nutrition on cortical morphology from childhood into adulthood.

## Supplementary Information

Below is the link to the electronic supplementary material.

Supplementary file1 (DOCX 34 KB)

Supplementary file1 (PDF 22 KB)

## Data Availability

Data used in this manuscript are not publicly available, but may be requested from the Director of Generation R Vincent Jaddoe (v.jaddoe@erasmusmc.nl), in accordance with local, national and European Union regulations.
