# Supplementary material for: The association between body mass index and brain morphology in children: a population-based study
Source: Brain Struct Funct. 2021 Jan 23;226(3):787–800. doi: 10.1007/s00429-020-02209-0 (PMC7981300; doi:10.1007/s00429-020-02209-0)
Supplement: Supplementary file 1 — Supplementary file1 (DOCX 34 KB) [file 429_2020_2209_MOESM1_ESM.docx]

| **Supplementary Table 1: ANOVA model fit comparison for gyrification and cortical thickness** | | | | | | |
| --- | --- | --- | --- | --- | --- | --- |
| **Measurement** | **Model** | **Polynomial** | **Res. Df** | **Df** | **F** | **Pr (>F)** |
| *Mean local Gyrification Index* | *Model 1* | Linear vs. Squared | 3,147 | 1 | 28.31 | 0.0000001 |
|  |  | Squared vs. Cubic | 3,146 | 1 | 1.98 | 0.16 |
|  | *Model 2* | Linear vs. Squared | 3,154 | 1 | 8.91 | 0.003 |
|  |  | Squared vs. Cubic | 3,153 | 1 | 2.51 | 0.11 |
|  | *Model 3* | Linear vs. Squared | 3,146 | 1 | 8.20 | 0.004 |
|  |  | Squared vs. Cubic | 3,145 | 1 | 2.59 | 0.11 |
| *Mean cortical thickness* | *Model 1* | Linear vs. Squared | 3,154 | 1 | 0.81 | 0.37 |
|  |  | Squared vs. Cubic | 3,153 | 1 | 3.38 | 0.07 |
|  | *Model 2* | Linear vs. Squared | 3,147 | 1 | 1.83 | 0.18 |
|  |  | Squared vs. Cubic | 3,146 | 1 | 3.21 | 0.07 |
|  | *Model 3* | Linear vs. Squared | 3,146 | 1 | 1.80 | 0.18 |
|  |  | Squared vs. Cubic | 3,145 | 1 | 3.22 | 0.07 |
| Note: Model 1 is adjusted for sex, age and handedness. Model 2 is additionally adjusted for education and ethnicity of the mother, maternal smoking and drinking during the pregnancy and the IQ of the child. Model 3 is additionally adjusted for behavior of the child. | | | | | | |
|  | | | | | | |

| **Supplementary table 2: Baseline characteristics median split subgroups** | | | | |  |
| --- | --- | --- | --- | --- | --- |
|  | n | BMI-SDS<-0.21 | n | BMI-SDS≥0.21 | |
| Age BMI measurement | 1,581 | 9.78 (0.32) | 1,579 | 9.82 (0.36) | |
| Age MRI measurement | 1,581 | 10.11(0.58) | 1579 | 10.16(0.6) | |
| BMI-SDS | 1,581 | -0.56 (0.57) | 1,579 | 1.08 (0.65) | |
| IQ | 1,359 | 104.47 (14.63) | 1,379 | 101.42(14.87) | |
| Sex |  |  |  |  | |
| Girl | 822 | 52% | 768 | 48.6% | |
| Boy | 759 | 47.9% | 811 | 51.4% | |
| Handedness |  |  |  |  | |
| Right | 1,376 | 87% | 1,375 | 87.1% | |
| Left | 158 | 10% | 156 | 9.9% | |
| Missing | 47 | 3% | 48 | 3% | |
| Ethnicity |  |  |  |  | |
| Dutch | 1,015 | 64.2% | 781 | 49.5% | |
| Other Western | 191 | 12.1% | 187 | 11.8% | |
| Non Western | 348 | 22.0% | 571 | 36.2% | |
| Missing | 27 | 1.7% | 40 | 2.5% | |
| Education Level |  |  |  |  | |
| High | 914 | 57.8% | 627 | 39.7% | |
| Middle | 492 | 31.1% | 682 | 43.2% | |
| Low | 67 | 4.2% | 120 | 7.6% | |
| Missing | 108 | 6.8% | 150 | 9.5% | |
| Alcohol use |  |  |  |  | |
| Never drank in pregnancy | 443 | 28% | 602 | 38.1% | |
| Drank until pregnancy was known | 190 | 12% | 177 | 11.2% | |
| Continued to drink in pregnancy occasionally | 550 | 34.8% | 419 | 26.5% | |
| Continued to drink in pregnancy frequently* | 146 | 9.2% | 100 | 6.3% | |
| Missing | 252 | 15.9% | 281 | 17.8% | |
| Smoking |  |  |  |  | |
| Never smoked during pregnancy | 1,104 | 69.8% | 1,034 | 65.5% | |
| Smoked until pregnancy was known | 131 | 8.3% | 115 | 7.3% | |
| Continued smoking in pregnancy | 163 | 10.3% | 207 | 13.1% | |
| Missing | 183 | 11.6% | 223 | 14.1% | |
|  |  |  |  |  | |
| Child Behavior Check List | 1,380 | 16.25 (14.16) | 1,306 | 17.54 (15.48) | |
| Note: Values are frequencies for categorical measures, means and standard deviations for continuous measures. | | | | |  |
| *Frequent continued alcohol use is defined as one or more glasses of alcohol per week in at least two trimesters. | | | | |  |

| **Supplementary table 3: the association between BMI-SDS and local cortical thickness** | | | | | | | |
| --- | --- | --- | --- | --- | --- | --- | --- |
| **Model** | **Hemisphere** | **Anatomical Region** | **Area Size (mm2)** | **MNI** | | | **Mean coefficient** |
|  |  |  |  | x | y | z |  |
| *Model 1* |  |  |  |  |  |  |  |
|  | RH | Inferior temporal | 2568.41 | 53.6 | -24.9 | -29.6 | 0.033 |
|  |  | Inferior parietal | 144`.10 | 38.5 | -68.5 | 45.6 | 0.022 |
|  |  | Postcentral | 1217.53 | 18.6 | -30.9 | 71.4 | 0.021 |
|  |  | Lingual | 1160.83 | 14.0 | -49.8 | -4.5 | -0.024 |
|  |  | Lateral occipital | 880.09 | 15.4 | -101.3 | 5.0 | 0.017 |
|  |  | Pericalcarine | 800.90 | 14.7 | -73.5 | 6.3 | -0.018 |
|  |  | Superior parietal | 386.70 | 19.0 | -74.2 | 44.4 | 0.021 |
|  |  | Superior parietal | 353.43 | 14.7 | -62.1 | 62.7 | 0.024 |
|  | LH | Lateral occipital | 4592.17 | -42.7 | -82.2 | -1.3 | 0.024 |
|  |  | Superior temporal | 2845.35 | -28.5 | -1.0 | -35.7 | 0.031 |
|  |  | Postcentral | 1156.24 | -12.8 | -35.4 | 75.1 | 0.023 |
|  |  | Lingual | 803.87 | -10.3 | -73.7 | -8.0 | -0.019 |
|  |  | Superior parietal | 608.09 | -22.8 | -58.0 | 64.1 | 0.026 |
|  |  | Cuneus | 329.06 | -11.8 | -68.5 | 14.0 | -0.017 |
| *Model 2* | RH | Lateral occipital | 3143.31 | 15.4 | -101.0 | 6.0 | 0.021 |
|  |  | Postcentral | 1873.50 | 18.6 | -30.9 | 71.4 | 0.021 |
|  |  | Superior temporal | 1482.61 | 45.0 | 11.7 | -24.3 | 0.030 |
|  |  | Lingual | 612.57 | 14.0 | -49.8 | -4.5 | -0.021 |
|  |  | Inferior temporal | 427.62 | 52.7 | -25.0 | -29.0 | 0.030 |
|  |  | Superior parietal | 395.81 | 20.4 | -74.9 | 44.1 | 0.021 |
|  |  | Pericalcarine | 316.61 | 13.5 | -72.4 | 6.1 | -0.019 |
|  | LH | Lateral occipital | 5183.47 | -17.5 | -101.8 | -4.1 | 0.024 |
|  |  | Superior temporal | 1981.61 | -28.5 | 0.2 | -36.6 | 0.028 |
|  |  | Postcentral | 1500.62 | -41.6 | -27.8 | 62.8 | 0.023 |
|  |  | Superior parietal | 624.68 | -23.6 | -57.0 | 64.2 | 0.024 |
|  |  | Lingual | 363.23 | -9.4 | -75.8 | -6.7 | -0.016 |
| *Model 3* | RH | Lateral occipital | 3147.39 | 15.4 | -101.0 | 6.0 | 0.021 |
|  |  | Postcentral | 1882.71 | 18.6 | -30.9 | 71.4 | 0.021 |
|  |  | Superior temporal | 1485.55 | 45.0 | 11.7 | -24.3 | 0.030 |
|  |  | Inferior temporal | 423.13 | 52.7 | -25.0 | -29.0 | 0.021 |
|  |  | Superior parietal | 399.17 | 20.4 | -74.9 | 44.1 | -0.018 |
|  |  | Lingual | 371.01 | 14.5 | -72.2 | -4.8 | -0.018 |
|  |  | Pericalcarine | 313.60 | 13.5 | -72.4 | 6.1 | -0.025 |
|  |  | Lingual | 225.57 | 14.0 | -49.8 | -4.5 | 0.023 |
|  | LH | Lateral occipital | 5195.22 | -17.5 | -101.8 | -4.1 | 0.024 |
|  |  | Superior temporal | 1981.67 | -28.1 | 0.5 | -36.9 | 0.028 |
|  |  | Postcentral | 1502.51 | -41.6 | -27.8 | 62.8 | 0.023 |
|  |  | Superior parietal | 624.26 | -23.6 | -57.0 | 64.2 | 0.024 |
|  |  | Lingual | 364.86 | -9.6 | -76.8 | -5.4 | -0.016 |
| *Model 4* | RH | Lingual | 1229.87 | 13.9 | -49.8 | -5.0 | -0.022 |
|  |  | Postcentral | 1128.85 | 18.6 | -30.9 | 71.4 | 0.019 |
|  |  | Caudal middle frontal | 962.59 | 34.3 | 14.6 | 32.8 | -0.016 |
|  |  | Lateral occipital | 848.22 | 16.2 | -101.1 | 4.4 | 0.018 |
|  |  | Pericalcarine | 757.05 | 14.1 | -72.9 | 6.2 | -0.018 |
|  |  | Lateral occipital | 573.68 | 45.5 | -81.1 | 2.2 | 0.018 |
|  |  | Superior temporal | 525.67 | 45.2 | 12.7 | -24.2 | 0.026 |
|  |  | Rostral middle frontal | 405.22 | 36.2 | 51.3 | 8.4 | -0.019 |
|  |  | Inferior temporal | 290.53 | 52.7 | -25.0 | -29.0 | 0.030 |
|  |  | Middle temporal | 261.59 | 64.5 | -30.0 | -10.1 | -0.020 |
|  |  | Middle temporal | 227.83 | 56.8 | -22.0 | -10.1 | -0.022 |
|  |  | Superior frontal | 214.81 | 9.2 | 58.4 | -21.0 | -0.024 |
|  |  | Superior frontal | 208.02 | 8.9 | 28.3 | 13.8 | -0.022 |
|  |  | Superior frontal | 201.65 | 10.7 | -0.4 | 56.2 | -0.022 |
|  | LH | Lateral occipital | 2730.23 | -17.4 | -101.6 | -4.9 | 0.023 |
|  |  | Rostral middle frontal | 1381.03 | -38.6 | 31.0 | 17.8 | -0.016 |
|  |  | Postcentral | 1049.9 | -41.9 | -27.4 | 62.6 | 0.021 |
|  |  | Lingual | 690.98 | -9.8 | -74.6 | -7.8 | -0.018 |
|  |  | Inferior temporal | 673.49 | -26.1 | 0.5 | -36.3 | 0.030 |
|  |  | Rostral middle frontal | 628.93 | -33.0 | 46.9 | 18.3 | -0.016 |
|  |  | Superior frontal | 520.42 | -10.3 | 16.9 | 60.3 | -0.023 |
|  |  | Superior temporal | 313.28 | -41.9 | 5.3 | -26.0 | 0.022 |
|  |  | Cuneus | 249.29 | -9.0 | -75.7 | 20.3 | -0.015 |
|  |  | Precentral | 240.06 | -21.6 | -15.3 | 58.8 | -0.018 |
|  |  | Superior parietal | 202.99 | -10.6 | -31.0 | 29.8 | -0.016 |
|  |  | Lateral orbitofrontal | 198.16 | -16.4 | 32.8 | -22.3 | -0.021 |
|  |  | Superior parietal | 196.21 | -23.1 | -57.0 | 64.1 | 0.022 |
| Note: Model 1 is adjusted for sex, age and handedness. Model 2 is additionally adjusted for education and ethnicity of the mother, maternal smoking and drinking during the pregnancy and the IQ of the child. Model 3 is additionally adjusted for behavior of the child. Model 4 is additionally corrected for mean cortical thickness Correction for multiple testing was performed using randomize. | | | | | | | |

| **Supplementary Table 4. Top 3 regions for each cluster with significant associations between BMI-SDS and lGI** | | | | | | | | | |
| --- | --- | --- | --- | --- | --- | --- | --- | --- | --- |
| Weight status | Model | Hemisphere | Annotated cluster | Top 1 region | % of cluster in top 1 region | Top 2 region | % of cluster in top 2 region | Top 3 region | % of cluster in top 3 region |
| Low | Model 1 | RH | Posterior cingulate | Paracentral | 47.8% | Posterior cingulate | 45.0% | Superior frontal | 7.2% |
|  |  | LH | Paracentral | Posterior cingulate | 49.5% | Paracentral | 38.4% | Precuneus | 12.1% |
| High | Model 1 | RH | Rostral middle frontal | Precentral | 11.5% | Rostral middle frontal | 10.6% | Postcentral | 9.8% |
|  |  |  | Precuneus | Precuneus | 45.7% | Lingual | 27.1% | Pericalcarine | 12.5% |
|  |  |  | Paracentral | Paracentral | 51.3% | Posterior cingulate | 39.8% | Precuneus | 9.0% |
|  |  | LH | Precentral | Precentral | 12.3% | Postcentral | 10.8% | Superior temporal | 10.7% |
|  |  |  | Rostral middle frontal | Rostral middle frontal | 26.8% | Superior frontal | 24.0% | Lateral orbitofrontal | 19.8% |
|  | Model 2 | RH | Rostral middle frontal | Rostral middle frontal | 80.2% | Pars orbitalis | 11.2% | Lateral orbitofrontal | 8.5% |
|  |  |  | Middle temporal | Middle temporal | 33.8% | Superior temporal | 33.5% | Banks of the superior temporal sulcus | 25.6% |
|  |  |  | Temporal pole | Temporal pole | 54.0% | Inferior temporal | 23.7% | Fusiform | 18.9% |
|  |  | LH | Precentral | Precentral | 45.0% | Postcentral | 35.4% | Supramarginal | 14.9% |
|  |  |  | Superior temporal | Superior temporal | 68.2% | Transverse temporal | 19.6% | Insula | 12.2% |
|  |  |  | Superior temporal | Superior temporal | 48.5% | Middle temporal | 36.7% | Banks of the superior temporal sulcus | 14.8% |
|  | Model 3 | RH | Rostral middle frontal | Rostral middle frontal | 80.5% | Pars orbitalis | 11.3% | Lateral orbitofrontal | 8.0% |
|  |  |  | Middle temporal | Middle temporal | 34.3% | Superior temporal | 34.2% | Banks of the superior temporal sulcus | 24.4% |
|  |  |  | Temporal pole | Temporal pole | 55.3% | Inferior temporal | 24.2% | Fusiform | 17.5% |
|  |  | LH | Postcentral | Precentral | 45.2% | Postcentral | 36.1% | Supramarginal | 14.4% |
|  |  |  | Superior temporal | Superior temporal | 69.0% | Transverse temporal | 20.0% | Insula | 11.0% |
|  | Model 4 | LH | Postcentral | Postcentral | 61.3% | Precentral | 38.7% |  |  |

| **Supplementary Table 5. Top 3 regions for each cluster with significant associations between BMI-SDS and CT** | | | | | | | | |
| --- | --- | --- | --- | --- | --- | --- | --- | --- |
| *Model* | *Hemi-sphere* | *Annotated cluster* | *Top 1 region* | *% of cluster in top 1 region* | *Top 2 region* | *% of cluster in top 2 region* | *Top 3 region* | *% of cluster in top 3 region* |
| 1 | RH | Inferior temporal | Superior temporal | 24.9% | Inferior temporal | 19.8% | Fusiform | 16.6% |
|  |  | Inferior parietal | Inferior parietal | 51.3% | Lateral occipital | 48.8% |  |  |
|  |  | Postcentral | Postcentral | 79.7% | Superior parietal | 11.5% | Precuneus | 8.8% |
|  |  | Lingual | Lingual | 97.4% | Parahippocampal | 2.6% |  |  |
|  |  | Lateral occipital | Lateral occipital | 99.0% | Pericalcerine | 1.0% |  |  |
|  |  | Pericalcarine | Pericalcerine | 57.8% | Cuneus | 32.7% | Lingual | 9.5% |
|  |  | Superior parietal | Superior parietal | 100.0% |  |  |  |  |
|  |  | Superior parietal | Superior parietal | 100.0% |  |  |  |  |
|  | LH | Lateral occipital | Lateral occipital | 50.3% | Inferior parietal | 33.6% | Superior parietal | 15.7% |
|  |  | Superior temporal | Superior temporal | 35.2% | Inferior temporal | 16.8% | Fusiform | 12.7% |
|  |  | Postcentral | Postcentral | 91.0% | Superior parietal | 5.6% | Precuneus | 2.3% |
|  |  | Lingual | Lingual | 94.0% | Pericalcerine | 3.0% | Parahippocampal | 3.0% |
|  |  | Superior parietal | Superior parietal | 100.0% |  |  |  |  |
|  |  | Cuneus | Cuneus | 52.9% | Pericalcerine | 47.1% | Cuneus | 0.5% |
| 2 | RH | Lateral occipital | Lateral occipital | 73.5% | Inferior parietal | 25.6% | Precuneus | 8.2% |
|  |  | Postcentral | Postcentral | 77.7% | Superior parietal | 10.0% | Fusiform | 23.2% |
|  |  | Superior temporal | Superior temporal | 32.3% | Insula | 23.3% |  |  |
|  |  | Lingual | Lingual | 100.0% |  |  |  |  |
|  |  | Inferior temporal | Inferior temporal | 100.0% |  |  |  |  |
|  |  | Superior parietal | Superior parietal | 100.0% |  |  |  |  |
|  |  | Pericalcarine | Pericalcerine | 86.5% |  |  |  |  |
|  | LH | Lateral occipital | Lateral occipital | 51.9% | Inferior parietal | 33.4% | Superior parietal | 14.6% |
|  |  | Superior temporal | Superior temporal | 36.0% | Inferior temporal | 17.7% | Fusiform | 16.3% |
|  |  | Postcentral | Postcentral | 88.7% | Precentral | 3.8% | Superior parietal | 3.7% |
|  |  | Superior parietal | Superior parietal | 100.0% |  |  |  |  |
|  |  | Lingual | Lingual | 95.9% | Pericalcerine | 4.1% |  |  |
| 3 | RH | Lateral occipital | Lateral occipital | 73.7% | Inferior parietal | 25.4% | Cuneus | 0.4% |
|  |  | Postcentral | Postcentral | 77.3% | Superior parietal | 10.2% | Precuneus | 8.3% |
|  |  | Superior temporal | Superior temporal | 32.3% | Insula | 23.3% | Fusiform | 23.2% |
|  |  | Inferior temporal | Inferior temporal | 100.0% |  |  |  |  |
|  |  | Superior parietal | Superior parietal | 100.0% |  |  |  |  |
|  |  | Lingual | Lingual | 100.0% |  |  |  |  |
|  |  | Pericalcarine | Pericalcerine | 86.2% | Lingual | 13.8% |  |  |
|  |  | Lingual | Lingual | 100.0% |  |  |  |  |
|  | LH | Lateral occipital | Lateral occipital | 51.9% | Inferior parietal | 33.4% | Superior parietal | 14.5% |
|  |  | Superior temporal | Superior temporal | 35.9% | Inferior temporal | 17.7% | Fusiform | 16.3% |
|  |  | Postcentral | Postcentral | 88.7% | Precentral | 3.9% | Superior parietal | 3.7% |
|  |  | Superior parietal | Superior parietal | 100.0% |  |  |  |  |
|  |  | Lingual | Lingual | 95.9% | Pericalcerine | 4.1% |  |  |
|  |  | Precentral | Precentral |  |  |  |  |  |
| 4 | RH | Lingual | Lingual | 96.3% | Fusiform | 1.6% | Parahippocampal | 1.5% |
|  |  | Postcentral | Postcentral | 92.8% | Superior parietal | 4.2% | Precentral | 2.0% |
|  |  | Caudal middle frontal | Precentral | 58.0% | Caudal middle frontal | 41.1% | Postcentral | 0.9% |
|  |  | Lateral occipital | Lateral occipital | 100.0% |  |  |  |  |
|  |  | Pericalcarine | Pericalcerine | 56.4% | Cuneus | 33.7% | Lingual | 10.0% |
|  |  | Lateral occipital | Lateral occipital | 98.7% | Inferior parietal | 1.3% |  |  |
|  |  | Superior temporal | Superior temporal | 48.9% | Insula | 40.0% | Temporal pole | 11.1% |
|  |  | Rostral middle frontal | Rostral middle frontal | 100.0% |  |  |  |  |
|  |  | Inferior temporal | Inferior temporal | 100.0% |  |  |  |  |
|  |  | Middle temporal | Middle temporal | 88.8% | Inferior temporal | 11.2% |  |  |
|  |  | Middle temporal | Middle temporal | 77.4% | Inferior temporal | 22.6% |  |  |
|  |  | Superior frontal | Superior frontal | 100.0% |  |  |  |  |
|  |  | Superior frontal | Superior frontal | 100.0% |  |  |  |  |
|  |  | Superior frontal | Superior frontal | 89.4% | Paracentral | 10.6% |  |  |
|  | LH | Lateral occipital | Lateral occipital | 89.1% | Inferior parietal | 7.6% | Superior parietal | 3.3% |
|  |  | Rostral middle frontal | Rostral middle frontal | 67.8% | Pars triangularis | 22.1% | Pars opercularis | 10.1% |
|  |  | Postcentral | Postcentral | 97.6% | Superior parietal | 1.8% | Paracentral | 0.4% |
|  |  | Lingual | Lingual | 92.0% | Pericalcerine | 7.4% | Parahippocampal | 0.5% |
|  |  | Inferior temporal | Inferior temporal | 41.7% | Fusiform | 29.4% | Entorhinal | 20.1% |
|  |  | Rostral middle frontal | Rostral middle frontal | 100.0% |  |  |  |  |
|  |  | Superior frontal | Superior frontal | 100.0% |  |  |  |  |
|  |  | Superior temporal | Superior temporal | 97.3% | Temporal pole | 2.7% |  |  |
|  |  | Cuneus | Cuneus | 83.2% | Pericalcerine | 16.8% |  |  |
|  |  | Precentral | Precentral | 85.8% | Superior frontal | 14.2% |  |  |
|  |  | Superior frontal | Superior frontal | 100.0% |  |  |  |  |
|  |  | Lateral orbitofrontal | Lateral orbitofrontal | 95.8% | Medial orbitofrontal | 4.2% |  |  |
|  |  | Superior parietal | Superior parietal | 100.0% |  |  |  |  |
