# Supplementary material for: The association between body mass index and brain morphology in children: a population-based study
Source: Brain Struct Funct. 2021 Jan 23;226(3):787–800. doi: 10.1007/s00429-020-02209-0 (PMC7981300; doi:10.1007/s00429-020-02209-0)
Supplement: Supplementary file 2 — Supplementary file1 (PDF 22 KB) [file 429_2020_2209_MOESM2_ESM.pdf]

Supplementary figure 1: Distribution of BMI-SDS

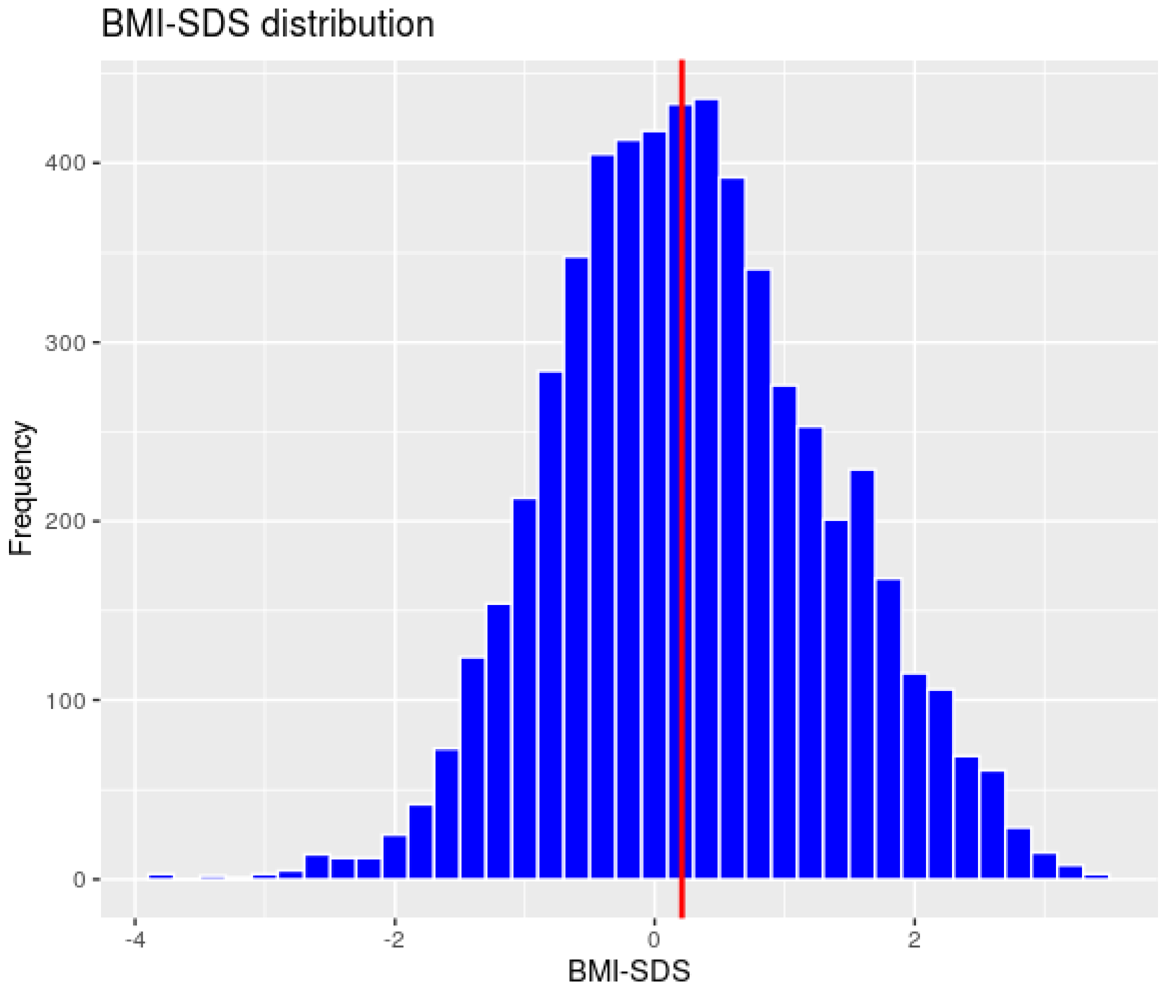

Note: the vertical red line shows the BMI-SDS median (BMI-SDS=0.21)
